# Impact of Occupational Exposures and Genetic Polymorphisms on Recurrence and Progression of Non-Muscle-Invasive Bladder Cancer

**DOI:** 10.3390/ijerph15081563

**Published:** 2018-07-24

**Authors:** Angela Carta, Sofia Pavanello, Giuseppe Mastrangelo, Ugo Fedeli, Cecilia Arici, Stefano Porru

**Affiliations:** 1 Department of Medical and Surgical Specialties, Radiological Sciences and Public Health, Section of Public Health and Human Sciences, University of Brescia, Brescia 25123, Italy; angela.carta@unibs.it; 2 University Research Center “Integrated Models for Prevention and Protection in Environmental and Occupational Health”, University of Brescia, Brescia 25123, Italy; cecilia.arici@univr.it (C.A.); stefano.porru@univr.it (S.P.); 3 Department of Cardiac, Thoracic, and Vascular Sciences, Unit of Occupational Medicine, University of Padova, Padova 35128, Italy; giuseppe.mastrangelo@unipd.it; 4 Epidemiological Department of the Veneto Region, Padova 35131, Italy; ugo.fedeli@azero.veneto.it; 5 Department of Diagnostics and Public Health, Section of Occupational Health, University of Verona, Verona 37134, Italy

**Keywords:** bladder cancer, non-muscle-invasive bladder cancer, recurrence, progression, occupational exposures, genetic polymorphisms, MnSOD, COMT

## Abstract

**Introduction:** Additional or better markers are needed to guide the clinical monitoring of patients with non-muscle-invasive bladder cancer (NMIBC). **Aim:** To investigate the influence of occupational exposures and genetic polymorphisms on recurrence and progression of NMIBC. **Methods:** The study includes 160 NMIBC patients. We collected on questionnaire information on demographic variables, lifetime smoking history, lifetime history of occupational exposure to aromatic amines and polycyclic aromatic hydrocarbons. Genetic polymorphism (glutathione S-transferase M1; T1; P1 (GSTM1; GSTT1; GSTP1); N-acetyltransferase 1; 2 (NAT1; NAT2); cytochrome P450 1B1 (CYP1B1); sulfotransferase 1A1 (SULT1A1); myeloperoxidase (MPO); catechol-O-methyltransferase (COMT); manganese superoxide dismutase (MnSOD); NAD(P)H:quinone oxidoreductase (NQO1); X-ray repair cross-complementing group 1; 3 (XRCC1; XRCC3) and xeroderma pigmentosum complementation group (XPD)) was assessed in peripheral blood lymphocytes. DNA adducts were evaluated by 32P-postlabeling. Predictors of recurrence (histological confirmation of a newly found bladder tumor) and progression (transition of tumor from low-grade to high-grade and/or increase in TNM stage) were identified by multivariate Cox proportional hazard regression with stepwise backward selection of independent variables. Hazard ratios (HR) with 95% confidence interval (95%CI) and two-tail probability of error (*p*-value) were estimated. **Results:** The risk of BC progression decreased with the homozygous genotype “ValVal” of both COMT and MnSOD (HR = 0.195; 95%CI = 0.060 to 0.623; *p* = 0.006). The results on BC recurrence were of borderline significance. No occupational exposure influenced recurrence or progression. **Conclusion:** Our results are supported by experimental evidence of a plausible mechanism between cause (ValVal genotype of both MnSOD and COMT) and effect (decreased progression of tumor in NMIBC patients). The genetic polymorphisms associated with better prognosis may be used in clinic to guide selection of treatment for patients initially diagnosed with NMIBC. However, external validation studies are required.

## 1. Introduction

### 1.1. Background

Approximately 75% of incident cases of bladder cancer (BC) present as non-muscle-invasive BC (NMIBC) and 25% as muscle-invasive BC (MIBC) [1]. Recurrence of NMIBC has been found as rapidly as in a few weeks after treatment/surgery [2]. The relapsing nature of NMIBC requires frequent clinical checks and repeated treatment [3,4]. Therefore, NMBIC represent a highly expensive disease for both patients and health care systems [5].

The standard conservative therapy—transurethral resection (TUR) of the bladder tumor combined with intravesical instillations with Bacillus Calmette-Guérin and/or intravesical chemotherapy (BCG)—can hinder recurrence, but the results in view to progression are inconsistent [6,7,8].

Whether intravesical instillations increase cancer-specific survival (CSS) is yet debated [8,9]. A too long conservative treatment in high-risk patients could postpone cystectomy, allowing progression to muscle-invasive disease [10,11,12]. For example, in a retrospective study, CSS at three years was 37% in the group with progression to MIBC compared to 67% in the group with primary MIBC [13]. These findings indicate that an early radical treatment could increase survival of patients with high-risk NMIBC [14] and in the meantime prompt the need of an early stage detection of these high-risk NMIBC patients.

It has been reported that the clinicopathological variables, used in the risk tables of European Organization for Research and Treatment of Cancer (EORTC), are not able to accurately predict the risk of disease recurrence and progression in individual patients [3]. Likewise, scores that include molecular markers have insufficient discriminative ability [15,16]. Additional or better prognostic tools are needed to guide clinicians in the management of patients with NMIBC [17].

There is an increasing evidence that genetic polymorphisms could have a role in estimating the prognosis of disease and response of treatment [18,19,20,21]. However, genetic investigations for BC prognosis are rare, of small size, and without independent replications to exclude false-positive results [22,23,24].

The influence of occupational exposure on BC prognosis was investigated in 794 bladder cancer patients from Germany. A shorter relapse-free survival, though not significant, was observed in some occupations including those entailing exposure to aromatic amines [25]. Likewise, in another study, no clear impact of occupational exposure was detected on recurrence nor relapse-free time. However, exposure to aromatic amines displayed a tendency to more relapses and shorter relapse-free times [26]. Therefore, these findings are worth further investigation.

Genome wide association studies show that genetic variants have relevance on both etiology and prognosis for a number of tumors, including colorectal, pancreatic, breast, lung, and prostate cancer [27,28,29,30,31,32,33], including BC [34]. 

### 1.2. Objectives

The aim of the present study is to investigate the extent to which the occupational exposures and genetic polymorphisms influence the recurrence and progression of the tumor in a group of NMIBC patients of a retrospective hospital based case-control study [35,36,37,38,39,40]. In particular, we inspect genetic polymorphisms of enzymes that are known from the literature [34,35,36,37,38,39,40] to be involved in metabolism and DNA damage repair of bladder carcinogens.

## 2. Methods

### 2.1. Study Design and Participants

The present study includes the “cases” arm stemming from an earlier hospital-based case-control study fully described in previous publications [35,36,37,38,39,40]. They are only newly diagnosed, histologically confirmed NMIBC patients. Out of 165 original subjects (all men), two were excluded because of immediate radical cystectomy and three because of missing data. 

During hospital admission (July 1997 to December 2000), information on demographic variables, lifetime smoking history, dietary habits, lifetime occupation history was collected by a trained interviewer through standardized questionnaire; blood samples were collected and on the same day processed by centrifugation for obtaining peripheral blood lymphocytes (PBLs). The Brescia ethical committee (i.e., internal review board) approved the study (ethical approval code: protocol number 2859/9185, 4 September 1996).

### 2.2. Genetic Polymorphisms

Automated DNA extraction was performed following the manufacturer’s instructions as previously described [35]. Genotyping of glutathione S-transferase M1 (GSTM1) null, GSTT1 null, GSTP1 I105V, *N*-acetyltransferase 1 (NAT1) fast, NAT2 slow, cytochrome P450 1B1 (CYP1B1) V432 L, sulfotransferase 1A1 (SULT1A1) R213H, myeloperoxidase (MPO) G-463A, catechol-*O*-methyltransferase (COMT) V108 M, manganese superoxide dismutase (MnSOD) A-9 V, NAD(P)H:quinone oxidoreductase (NQO1) P187S, X-ray repair crosscomplementing group 1 (XRCC1) R399Q, XRCC3 T241 M, and xeroderma pigmentosum complementation group (XPD) K751Q polymorphisms was assessed using Amplification Refractory Mutation System assay and using the GeneAmp PCR System 9700 (Applied Biosystems, Milano, Italy). PCR were followed by enzymatic digestion and PCR-RFLP analysis, as previously described [35,36,37,38,39,40]. In our study population the allele frequency of MPO G-463A, SULT1A1 Arg213His, GSTP1 Ile105Val, Ala114Val, MnSOD Ala-9Val, COMT Val108Met, XRCC1 Arg399Gln, XRCC3 Thr241Met, XPD Lys751Gln, NQO1 Pro187Ser, NAT1*10, *11 and NAT2*5A, *6A, *7A were 0.26, 0.22, 0.69, 0.38, 0.44, 0.45, 0.33, 0.41, 0.33, 0.24, 0.25, 0.89, 0.34, 0.62 and 0.70, respectively. Hardy -Weinberg Equilibrium (HWE) was tested for each polymorphism; the allele frequency was calculated and the observed genotype frequency was compared with expected frequency using x 2 test. The allele distributions for the polymorphisms were under HWE with *p*-value > 0.05. The polymorphisms were dichotomized according to additive genetic models on the basis of a priori evidence and on previous studies conducted on this study population. Genetic biomarkers were coded as 0/1 variables as follows: GSTM1 (“NULL” variant = 1, otherwise = 0); GSTP1 (“1A/ 1A” = 0, otherwise = 1); GSTT1 (“NULL” = 1); NAT1 (“S” = 1); NAT2 (“S” = 1); SULT (“1A1/1A1 = 1); MPO (“A/A” = 1); COMT (“WW” = 0); MnSOD (“WW” = 1); NQO1 (“MM” = 1); CYP1B1 (“WW” = 0); XRCC1 (“G/G” = 0); XRCC3 (“C/C” = 0); XPD (“A/A” = 0). The genotype frequency of MPO (“A/A” = 1); SULT (“1A1/1A1 = 1); GSTM1 (“NULL” variant = 1, otherwise = 0); GSTP1 (“1A/ 1A” = 0, otherwise = 1); GSTT1 (“NULL” = 1); MnSOD (“WW” = 1); COMT (“WW” = 0); XRCC1 (“G/G” = 0); XPD (“A/A” = 0); XRCC3 (“C/C” = 0); NQO1 (“MM” = 1); NAT1 (“S” = 1); NAT2 (“S” = 1) were 0,04; 0.04; 0.64; 0.52; 0.22; 0.22; 0.20; 0.11; 0.19; 0.12; 0.06; 0.69; 0.60, respectively. 

### 2.3. Occupational Exposures

Occupational exposures to polycyclic aromatic hydrocarbons (PAHs) and aromatic amines (AAs) were estimated as previously described [39]. Two dichotomous variables were coded for occupational exposure to PAHs and AAs being absent or present.

### 2.4. DNA Adducts

Aliquots of 5 μg DNA were assayed for the presence of bulky-DNA adducts by ^32^P-postlabeling after enrichment with Nuclease P1 as previously described [41,42]. Resolution of DNA adducts was performed by multidirectional thin-layer chromatography (TLC), using polyethyleneimine (PEI)-cellulose plates [43]. A 0/1 variable was coded when DNA adducts were lower/equal or higher than the 75° percentile. DNA adducts were measured by nuclease P1 32P-postlabeling, a non-specific method applicable to the detection and measurement of aromatic or bulky non-aromatic DNA adducts. In our earlier study [39], DNA adducts (*p* = 0.028) were found to be positively associated with occupational AAs exposure but not with BC risk. Therefore DNA adducts were likely biomarkers of exposure. However, the responsible electrophilic chemical could not be identified because adducts detected by the nuclease P1 method of 32P–post-labeling are not specific.

### 2.5. Other Predictors

Age at the first TUR biopsy was broken down in three classes: <60; 60–69; and ≥70 years. Smoking was categorized in three levels: current smokers; former smokers; nonsmokers.

### 2.6. Definition of Outcomes

Time to first recurrence of bladder cancer was the time elapsed between date of diagnosis and date of histological confirmation of a newly found bladder or prostatic urethra tumor following at least one tumor-negative follow-up cystoscopy or two surgical resection sessions for the primary tumor. Furthermore, a dichotomous variable was coded for presence/absence of recurrence.

Time to first progression of bladder cancer was the time elapsed between data of diagnosis and date of a recurrence at which one or more of the following events occurred:

- transition from low-grade (i.e., WHO 1973 differentiation grade 1 or 2) to high-grade (i.e., WHO 1973 differentiation grade 3);
- increase in T stage (i.e., from pTa/pTis to ≥pT1; or from pT1 to ≥pT2);
- increase in N stage (i.e., from N0 to ≥N1);
- increase in M stage (i.e., from M0 to M1);
- cystectomy for therapy-resistant or “uncontrollable” disease (no case).

Additionally, a dichotomous variable was coded for presence/absence of progression. We coded a dichotomous variable which was 0 (low grade tumor = TNM stage Ta in combination with WHO 1973 differentiation grade 1 or 2, WHO/ISUP 2004 low grade, or Malmstrom grade 1 or 2a) or 1 (high grade tumors = all other tumors). The variable did not have a significant effect in two models of Cox Proportional Hazard for BC progression and for BC recurrence (data not shown).

### 2.7. Statistical Methods

The distribution of patient characteristics (genetic polymorphisms, occupational exposure to PAHs or AAs, DNA adducts, age, smoking habits) by recurrence or progression status was separately analyzed with the chi-square test, setting at 0.05 the threshold of significance. 

The presence of an interaction between occupational exposure and adducts/polymorphisms associated to bladder cancer prognosis was never significant. The likelihood ratio tests were:

Chi-Square (1d.f.) = 0.04, *p* = 0.8446 − Adducts × exposure to PAH, bladder cancer recurrence

Chi-Square (1d.f.) = 1.00, *p* = 0.3177 − Adducts × exposure to aromatic amines, 

Chi-Square (1d.f.) = 0.06, *p* = 0.8033 − COMT × exposure to PAH, progression

Chi-Square (1d.f.) = 1.90, *p* = 0.1681 − COMT × exposure to aromatic amines, progression

Chi-Square (1d.f.) = 0.16, *p* = 0.6895 − MnSOD × exposure to PAH, progression

Chi-Square (1d.f.) = 0.72, *p* = 0.3967 − MnSOD × exposure to aromatic amines, progression.

Kaplan–Meier curves were plotted for the favorable genotypes, and log rank test was applied to compare the difference between the event free survival times of each genotype. To identify significant predictors for recurrence and progression we used the multivariate Cox proportional hazard regression analysis with stepwise backward selection of independent variables. The program estimates the hazard ratio (HR) with 95% confidence interval (95%CI) and two-tail probability of error (*p*-value). The statistical power of one regression coefficient in a Cox Proportional Hazard model, holding constant the coefficients of the other covariates, was computed through a STATA command by specifying the sample size (=160 patients), the effect size (the hazard ratio supplied by the model) and the overall probability of failure (=42/160 = 0.26 for BC progression).

The statistical analyses were performed with Stata 13.

## 3. Results

One hundred and sixty subjects (all men) were included in the analysis and followed up; the follow-up time was 4.63 ± 2.65 years. Using the HR provided by the model, the estimated power was 0.9996 for the combined homozygous polymorphisms of MnSOD and COMT.

### 3.1. Descriptive Data

Table 1 shows the cross tabulation of recurrence and progression. The number of cases was 90 (56%) and 42 (26%) for, respectively, recurrence and progression. The latter 42 patients also showed recurrence; the two clinical endpoints were not each other independent.

Table 2 shows the main characteristics of patients without/with recurrence as well as the *p*-value of the chi-square test. A significant result was obtained for NQO1 (*p* = 0.005) and DNA Adducts (*p* = 0.019). The disease prognosis was better (i.e., percentage of recurrence was lesser) for the homozygous genotype “MM” of NQO1. 

**Figure 1:**
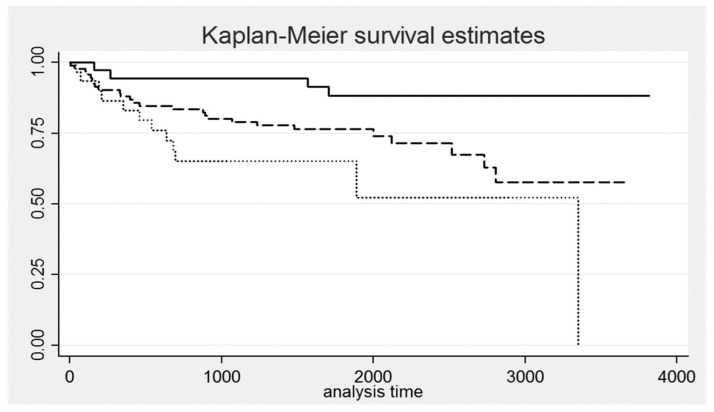
Kaplan-Meier survival curves for groups of patients with different combinations of homozygous genotypes of manganese superoxide dismutase (MnSOD) and catechol-*O*-methyltransferase (COMT) (none = dot line; one single = dash line; both = solid line).

Table 3 shows the main characteristics of patients without/with progression as well as the *p*-value of the chi-square test. Borderline significant results were obtained for MnSOD (*p* = 0.049), COMT (*p* = 0.084), NQO1 (*p* = 0.065) and DNA Adducts (*p* = 0.068). The disease prognosis was better (i.e., percentage of progression was lesser) for the homozygous genotypes: “WW” for COMT; “WW” for MnSOD; “MM” for NQO1. MnSOD and COMT were combined in a three-class variable. With none, one single and both homozygous polymorphisms, the cases of progression were, respectively, 12 (39%), 26 (27%) and 4 (11%); the chi-square for trend was 6.69 (*p* = 0.0097). The different risk of progression was graphically represented by the Kaplan-Meier survival curves. As shown in Figure 1, the prognosis was better for patients with both homozygous genotypes; these differences were significant at the log-rank test for equality of survivor functions (Chi^2^ = 9.73; degrees of freedom = 2; *p*-value = 0.0077). 

### 3.2. Outcome Data

Time to first recurrence of bladder cancer was in average 1131 days (about three years) with a standard deviation of 819 days. Table 4 shows the results of multivariable Cox regression analysis of recurrence after stepwise backward selection of predictor variables. It can be seen that the risk of bladder cancer recurrence increased with increasing age (HR = 1.020; 95%CI = 1.001 to 1.039; *p* = 0.036) and level of DNA adducts (HR = 1.655; 95%CI = 1.051 to 2.606; *p* = 0.030). 

Table 5 shows two models of Cox regression analysis of recurrence (HR, 95%CI and *p*-value) including only one predictor: exposure to PAHs (Model 1) or AAs (Model 2). Although no result was statistically significant, exposure to AAs and to a lesser extent to PAHs seem to increase the risk of recurrence. 

Time to progression of bladder cancer was in average 1690 days (about 4.6 years) with a standard deviation of 966 days. After stepwise backward selection of predictor variables, Table 6 shows the results of multivariable Cox regression analysis of progression. It can be seen that the risk of bladder cancer progression decreased with the homozygous genotype “WW” of COMT (HR = 0.482; 95%CI = 0.259 to 0.899; *p* = 0.022) and the homozygous genotype “WW” of MnSOD (HR = 0.446; 95%CI = 0.212 to 0.936; *p* = 0.033). 

MnSOD and COMT were combined in a three-class variable, which was coded as 0 (none), 1 (one or another) and 2 (both homozygous polymorphisms). Table 7 shows the results of multivariable Cox regression analysis of progression; it can be seen that only the combined variable entered the model after stepwise backward selection of independent variables. Using as reference the level 0, the level 2 showed the highest degree of protection (HR = 0.194; 95%CI = 0.060 to 0.629; *p* = 0.006). A backward stepwise selection was applied to identify polymorphisms possibly associated to the risk of recurrence/progression. Thereafter, for bladder cancer progression, an interaction could be observed between the two selected polymorphisms; the *p*-value = 0.006 observed for carriers of both homozygous polymorphisms (Table 7) corresponds to a False Discovery Rate below 10%; anyway study results need to be confirmed by independent research.

Table 8 shows two models of Cox regression analysis for progression including only one predictor: exposure to PAHs (Model 1) or AAs (Model 2). Although no result was statistically significant, exposure to AAs and to a lesser extent to PAHs seem to decrease the risk of progression.

## 4. Discussion

### 4.1. Key Results

Homozygous genotype “ValVal” of both MnSOD and COMT decreased the risk of progression of NMIBC. Increasing age and level of DNA adducts increased the risk of recurrence.

### 4.2. Strengths and Limitations

Strengths of this research pertain to the comprehensive and specific assessment of environmental and occupational bladder cancer risk factors, with a thorough assessment of major genetic polymorphisms related to bladder cancer as well as determination of some biomarkers of biological effect.

An obvious limitation of the present study is a sample size that might not be large enough to detect weaker relationships or associations based on few cases of recurrence and progression (see NQO1 in Table 2 and Table 3). 

In the present study, information on number of neoplastic foci and size of tumor was lacking. The latter are however two out of six clinical features of disease (i.e., tumor number, tumor size, frequency of prior recurrence, TNM stage, histologic grade and presence of carcinoma in situ) that are used in the EORTC Genito-Urinary Group tables to predict the probabilities of recurrence or progression of NMIBC to MBIC [44]. The European Association of Urology (EAU) has adopted these tables in its treatment guidelines, adapting the treatment to the risk of recurrence and progression of NMIBC patients [45]. 

The above missing information could be a possible limitation because it might affect the final analysis in terms of adjustment for potential covariates. It is worthy to note that, generally, tumor focality and size are poorly documented in medical charts [34]. Although tumor size was unknown for 995 (78%) out of 1269 NMIBC patients, authors nonetheless presented effect estimates on recurrence and progression of bladder cancer for 12 genetic susceptibility variants using the univariable Cox proportional hazard regression [34]. In a study collecting data on both inflammatory-related genetic variants and clinic-pathologic variables, results evidenced a joint effect. In this study, carried out in 822 NMIBC patients followed-up >10 years, single nucleotide polymorphisms (SNPs) appeared to complement the conventional prognostic methods based on pathologic variables, improving the discriminative ability of models that predict the clinical outcomes of non-muscle-invasive bladder cancer [17]. On the other hand, several studies done in the last decades have found a correlation between polymorphism in genes modulating oxidative stress and cancer susceptibility (see next section: Interpretation), suggesting that host genetic context can be an independent predictor. 

### 4.3. Interpretation

A statistical model seeks only to best describe the data. Below, we specify the relationships between the variables in terms of the biological processes that are thought to have given rise to the data. 

#### 4.3.1. Manganese Superoxide Dismutase 

In all mammalian cells the aerobic respiration inevitably produces reactive oxygen species (ROS); the latter can be induced also by ionizing radiation, polycyclic aromatic hydrocarbons and smoking [46]. MnSOD is the only mitochondrial enzyme that dismutases the superoxide anion (O_2_^−^) [47], which primarily arises from electron escape in the mitochondrial electron transport chain [48]. Following dismutation by MnSOD, the resultant hydrogen peroxide (H_2_O_2_) is a more stable ROS compared to O2− and can easily diffuse throughout the cell, where it may be further processed by catalase (CAT) or glutathione peroxidase (GPx) to O_2_ and H_2_O [49].

A polymorphism encoding for either valine (Val) or alanine (Ala) at codon 16 has been described in the human MnSOD gene [50]. Ala is the mutant allele and Val is the wild-type allele; ValVal is the wild-type (WW) MnSOD genotype. 

The role of MnSOD and its changes in gene expression during tumorigenicity in several cancer cell lines have been areas of intense investigation in the last decades; these studies have been reviewed in recent years [48,49,51]. In human bladder cancer, however, such information is quite limited. 

According to Hempel [52], publicly available expression data from cancer microarrays (source: oncomine.org) indicate that mitochondrial MnSOD expression was consistently elevated in high grade tumor specimens and in invasive bladder tumors. Conversely, levels of catalase remained mostly unchanged, except in one study where catalase expression showed a statistically significant decrease in invasive bladder cancer compared to superficial bladder cancer specimen [52].

A highly metastatic line (253JB-V) has been obtained from human transitional BC cells [53]. Relative to the activity of their 253J parental cell lines, this metastatic cell model showed a pronounced increase of cellular MnSOD activity, while the activity of catalase was significantly reduced and glutathione peroxidase remained relatively unaffected [52]. Given the enhanced dismuting activity of MnSOD and the decreased levels of catalase, compared to 253J cells, 253J B-V cells showed an approximately 1.5 fold increase in H_2_O_2_ production. Expression of pro-metastatic and pro–angiogenic factors (matrix metalloproteinase 9 and vascular endothelial derived growth factor) were upregulated in the metastatic line and shown to be H_2_O_2_-dependent [52]. 

On the other hand, a study was performed on paraffin-embedded tissues (obtained from 75 bladder cancers and 30 normal bladders) that were treated with immunohistochemical staining for catalase, superoxide dismutase and glutathione peroxidase. With respect to superficial transitional cell carcinomas, catalase and MnSOD expression was significantly lower in invasive transitional cell carcinomas, thus appearing to be associated with progression of BC [54].

Worthy to notice, in several types of cancer, the ratios MnSOD/catalase and MnSOD/glutathione peroxidase have been suggested as biomarkers for tumor progression and metastasis [55].

While the above-cited literature took into account the level of gene expression, our work considers the polymorphism, which causes replacement of alanine with valine at amino acid 16 (Ala16Val) [56]. The polymorphism, by altering the secondary structure of MnSOD, leads to defective mitochondrial localization. The MnSOD with Val allele was found to be 30–40% less active than the wild-type MnSOD [57]. 

As reported by Hung [36] and confirmed by Porru [39] in the case-control study from which the present bladder cancer patients were obtained, ValVal MnSOD genotype was a risk factor for bladder cancer. Thus, ValVal MnSOD genotype while improving the prognosis (by reducing the progression of NMIBC toward MIBC) increased the risk of bladder cancer. As mitochondrial MnSOD dismutases the superoxide anion (O_2_^−^) into H_2_O_2_, any decrease in activity in MnSOD tends to increase O_2_− and decrease H_2_O_2_ (Equation (1) below), with varying and contrasting effects depending on cancer type and stage of the disease.

(1) $$O_{2}^{-}\overset{\mathrm{MnSOD}}{\to }H_{2}O_{2}\overset{\mathrm{CAT}\;\&\;{\mathrm{CP}}_{X}}{\to }H_{2}O$$

In our investigation, we postulate that O_2_^−^ damages DNA (resulting in bladder cancer initiation) while H_2_O_2_ promotes tumor proliferation and metastasis via pro-metastatic and pro–angiogenic factors (matrix metalloproteinase 9 and vascular endothelial derived growth factor), leading to bladder cancer progression. 

#### 4.3.2. Catechol-*O*-methyltransferase

COMT is an enzyme that catalyzes the transfer of a methyl-group from S-adenosylmethionine to a catechol-containing substrate molecule. By catalyzing the methylation of various endobiotic and xenobiotic substances, COMT might protect DNA from damage [58]. In 1995 Lotta et al [59] identified in the COMT gene (at codon 108) a guanine (G) to adenine (A) transition that leads to the aminoacid substitution from valine (Val) to methionine (Met). The amino acid change involves a change in COMT activity; the enzyme activity of the MetMet genotype is a quarter of that of the wild genotype, while heterozygous subjects show intermediate enzyme activity [59]. 

Data about the relationship between COMT polymorphism and bladder cancer risk are limited. No statistically significant effects of the COMT genotypes on bladder tumorigenesis were observed among men from Italy [36,39] or France [60]. However, Wolpert [61] showed an association between the low- or intermediate activity COMT genotypes (MetMet or ValMet) and decreased susceptibility to bladder cancer among Egyptian men and premenopausal women. To our knowledge, there are no studies reporting on the relationship between COMT and progression of NMIBC. Studies on association between COMT genotypes and various malignancies have shown inconsistent findings, indicating that additional interactions (gene–gene and gene–environment interactions) might modulate the role of the COMT [58]. Likewise, in our study it was the interaction of COMT (ValVal genotype) with MnSOD (ValVal genotype) rather than COMT alone that showed a significant impact, protecting from progression to muscle-invasive bladder cancer. 

## 5. Conclusions

Homozygous genotype “ValVal” of both MnSOD and COMT decreased the risk of progression of NMIBC. The results are supported by experimental evidence of a plausible mechanism. The genetic polymorphisms associated with better prognosis may be used in clinic to guide selection of treatment for patients initially diagnosed with NMIBC. Before, however, external validation studies are required. 

## Figures and Tables

**Table 1:** Cross tabulation of recurrence and progression.

		Progression		
		Yes	No	Total
Recurrence	Yes	42	48	90
	No	0	70	70
	Total	42	118	

**Table 2:** Main characteristics of patients without/with recurrence and *p*-values of the chi-square test.

Risk Factors	Classes	*n*	%	Without Recurrence	With Recurrence	% With Recurrence	*p* (Chi2)
Age (years)	<60	63	39.4%	30	33	52%	0.720
	60–69	47	29.4%	19	28	60%	
	≥70	50	31.3%	21	29	58%	
Smoking status	Current	79	49.4%	33	46	58%	0.250
	Former	69	43.1%	29	40	58%	
	Never	12	7.5%	8	4	33%	
Tumor aggressiveness	Low	41	25.6%	15	26	63%	0.284
	High	119	74.4%	55	64	54%	
MPO	0	154	96.3%	67	87	56%	0.753
	1	6	3.8%	3	3	50%	
SULT	0	153	95.6%	66	87	57%	0.465
	1	7	4.4%	4	3	43%	
GSTM1	0	57	35.6%	26	31	54%	0.724
	1	103	64.4%	44	59	57%	
GSTP1	0	76	47.5%	30	46	61%	0.300
	1	84	52.5%	40	44	52%	
GSTT1	0	125	78.1%	53	72	58%	0.515
	1	35	21.9%	17	18	51%	
MnSOD	0	106	66.3%	43	63	59%	0.255
	1	54	33.8%	27	27	50%	
COMT	0	48	30.0%	22	26	54%	0.728
	1	112	70.0%	48	64	57%	
XRCC1	0	72	45.0%	30	42	58%	0.631
	1	88	55.0%	40	48	55%	
XPD	0	58	36.3%	23	35	60%	0.431
	1	102	63.8%	47	55	54%	
XRCC3	0	72	45.0%	29	43	60%	0.423
	1	88	55.0%	41	47	53%	
NQO1	0	151	94.4%	62	89	59%	0.005
	1	9	5.6%	8	1	11%	
NAT1	0	110	68.8%	48	62	56%	0.966
	1	50	31.3%	22	28	56%	
NAT2	0	64	40.0%	26	38	59%	0.515
	1	96	60.0%	44	52	54%	
DNA Adducts	≤5.6	123	76.9%	60	63	51%	0.019
	>5.6	37	23.1%	10	27	73%	
Exposure to PAH	Never	100	62.5%	44	56	56%	0.934
	Ever	60	37.5%	26	34	57%	
Exposure to aromatic amines	Never	148	92.5%	66	82	55%	0.449
	Ever	12	7.5%	4	8	67%	

**Table 3:** Main characteristics of patients without/with progression and *p*-values of the chi-square test.

Risk Factors	Classes	Without Progression	With Progression	% With Progression	*p* (Chi^2^)
Age (years)	<60	49	14	22%	0.320
	60–69	36	11	23%	
	≥70	33	17	34%	
Smoking status	Current	60	19	24%	0.789
	Former	49	20	29%	
	Never	9	3	25%	
Tumor aggressiveness	Low	30	11	25%	0.922
	High	88	31	25%	
MPO	0	115	39	25%	0.178
	1	3	3	50%	
SULT	0	111	42	27%	0.106
	1	7	0	0%	
GSTM1	0	39	18	32%	0.254
	1	79	24	23%	
GSTP1	0	56	20	26%	0.986
	1	62	22	26%	
GSTT1	0	93	32	26%	0.724
	1	25	10	29%	
MnSOD	0	73	33	31%	0.049
	1	45	9	17%	
COMT	0	31	17	35%	0.084
	1	87	25	22%	
XRCC1	0	55	17	24%	0.493
	1	63	25	28%	
XPD	0	45	13	22%	0.406
	1	73	29	28%	
XRCC3	0	51	21	29%	0.448
	1	67	21	24%	
NQO1	0	109	42	28%	0.065
	1	9	0	0%	
NAT1	0	82	28	25%	0.734
	1	36	14	28%	
NAT2	0	46	18	28%	0.660
	1	72	24	25%	
DNA Adducts	≤5.6	95	28	23%	0.068
	>5.6	23	14	38%	
Exposure to PAH	Never	71	29	29%	0.307
	Ever	47	13	22%	
Exposure to aromatic amines	Never	109	39	26%	0.918
	Ever	9	3	25%	

**Table 4:** Results of multivariable Cox regression analysis for recurrence (hazard ratio (HR), 95% confidence interval (95%CI) and *p*-value) after stepwise backward selection of predictors.

	HR	95%CI	*p*-Value
Age (years)	1.020	1.001 to 1.039	0.036
DNA adducts ^@^	1.655	1.051 to 2.607	0.030

^@^ Dichotomous variable (lower/equal or higher than the 75° percentile).

**Table 5:** Two models of Cox regression analysis for recurrence (hazard ratio (HR), 95% confidence interval (95%CI) and *p*-value) including only occupational exposure to PAHs (Model 1) or aromatic amines (Model 2).

Occupational Exposure to:	HR	95%CI	*p*-Value
Model 1			
PAHs ^§^	1.077	0.701 to 1.654	0.735
Model 2			
Aromatic amines ^§^	1.129	0.743 to 1.743	0.569

^§^ Dichotomous variables: occupational exposure absent or present.

**Table 6:** Results of multivariable Cox regression analysis for progression (hazard ratio (HR), 95% confidence interval (95%CI) and *p*-value) after stepwise backward selection of predictors.

Genotypes	HR	95%CI	*p*-Value
COMT	0.482	0.259 to 0.899	0.022
MnSOD	0.446	0.212 to 0.936	0.033

**Table 7:** Results of multivariable Cox regression analysis for progression (hazard ratio (HR), 95% confidence interval (95%CI) and *p*-value) after stepwise backward selection of predictors. The dependent variable is a three-class variable with none (reference), one single or both homozygous polymorphisms of MnSOD and COMT.

Homozygous Polymorphisms of MnSOD & COMT	HR	95%CI	*p*-Value
None	1.00		
One single	0.571	0.275 to 1.185	0.132
Both	0.194	0.060 to 0.629	0.006

**Table 8:** Two models of Cox regression analysis for recurrence (hazard ratio (HR), 95% confidence interval (95%CI) and *p*-value) including only occupational exposure to PAHs (Model 1) or aromatic amines (Model 2).

Occupational Exposure to:	HR	95%CI	*p*-Value
Model 1			
PAHs ^§^	0.690	0.358 to 1.327	0.266
Model 2			
Aromatic amines ^§^	0.719	0.382 to 1.351	0.305

^§^ Dichotomous variables: occupational exposure absent or present
